# Prolonged drought regulates the silage quality of maize (*Zea mays* L.): Alterations in fermentation microecology

**DOI:** 10.3389/fpls.2022.1075407

**Published:** 2022-12-09

**Authors:** Xuejing Zi, Wan Wang, Shiyong Zhou, Feng Zhou, Dongyun Rao, Peng Shen, Siyang Fang, Bozhi Wu

**Affiliations:** ^1^ College of Agronomy and Biotechnology, Yunnan Agricultural University, Kunming, Yunnan, China; ^2^ Kunming Seed Management Station, Kunming, Yunnan, China

**Keywords:** drought stress, silage maize, nutritional and fermentation quality, *Lactobacillus*, lactic acid

## Abstract

Prolonged drought stress caused by global warming poses a tremendous challenge to silage production of maize. Drought during maize growth and development resulted in altered micro-environment for silage fermentation. How fermentation of silage maize responds to moisture scales remains uncharted territory. In this research, Maize water control trials were conducted and the silage quality and microbial community of drought-affected maize were determined. The results showed that drought stress significantly reduced the dry matter but increased root-to-shoot ratio, soluble sugar and malonaldehyde content in maize. Before fermentation, the crude protein, crude ash and acid detergent fiber contents were significantly increased but the ether extract content was decreased under drought. The crude protein and acid detergent fiber were significantly decreased in the drought affected group after fermentation. Furthermore, water stress at maize maturity stage greatly reduced the number of total bacteria in silage fermentation but increased the proportion of the lactobacillus and lactic acid content of silage. Drought stress alters the microbial ecosystem of the fermentation process and reconstitutes the diversity of the bacterial community and its metabolites. This study provides a theoretical basis for the study of changes in silage fermentation as affected by abiotic stresses.

## 1 Introduction

Maize (*Zea mays* L.), a specialty gramineous crop, is a good silage species and meets the needs of livestock development (Wang et al., 2016). Silage fermentation technology can be used to prolong the green-holding capacity of maize. As seasonal animal feed security, fermented silage still retains sufficient nutrients (Mauser et al., 2015). Currently, traditional rainfed agriculture has suffered shocks with crop yield and quality due to phased prolonged droughts triggered by global warming. The production of maize and silage fermentation have been adversely affected by drastic changes in water scales (Zi et al., 2022).

Drought stress is one of the key abiotic stress factors. The temporal and spatial heterogeneity of water changes the plant development process and the ordinary physiological and metabolic functions of its cells. Under drought stress, the abscisic acid (phytohormone) can participate in plant cell signaling and promote leaf stomatal closure, thereby reducing water loss (Eisenach et al., 2017). In addition, changes in organic matter redistribution in various plant organs, such as increased root’s growth, are effective ways to avoid drought injury (Karcher et al., 2008). Osmoregulation and antioxidation of cells are considered effective measures for plants to resist drought stress by synthesizing an excess of specific metabolites. A large increase in water-soluble carbohydrates elevates the cytosol concentration, thus, reduces the reverse water flow (Signori-Müller et al., 2021). Moreover, the overexpression of drought-response proteins leads to an upregulation of the crude protein percentage (Sahid et al., 2020). Cellulose is the main polysaccharide compound that is more difficult to degrade during fermentation or feeding (Zhao et al., 2019). Some studies confirm that drought can alter cellulose. For example, drought stress reduced the final fiber length in cotton (Wenqing et al., 2019). The physiological and nutritional parameters of the crop change drastically under moisture stress. Such changes may lead to further alterations in silage quality and fermentation microorganisms.

In numerous plant studies, drought can significantly regulate the moisture and soluble carbohydrate content in plants. Meanwhile, water and soluble sugars are both critical sources of survival for microorganisms. Small molecular sugars such as glucose, fructose, and sucrose are available for direct use by microorganisms (Liang et al., 2022). The fermentative microbial network shaped by drought is obviously distinct from the plant environment under good water conditions. Water is an effective tool to modify nutrient availability, plant physiological processes and microbial communities (Tan et al., 2021). The moisture content regulates the microbial abundance (Gao et al., 2022). In addition, fungus is more likely to colonize and develop at higher moisture levels. The environment can significantly affect bacterial and fungal communities (Wu et al., 2022). Water stress changes the nutrients and water in fermentation system, drought-stricken maize material indirectly alters the microbial society in fermentation.

Plant metabolites under drought are directly linked to the micro silage fermentation ecosystem. Composed of plants, endophytic bacteria, and plant metabolites, the entire anaerobic fermentation process is considered to be a miniature closed ecosystem (Brusetti et al., 2006). In these micro-ecosystems, lactobacillus are the main decomposers in fermentation, exchanging material and energy, and maintaining short-lived and continuous stability.

In this research, the physiological characteristics of drought-affected maize, the nutritional quality parameters before and after fermentation were determined through a water control experiment. In addition, the silage quality and changes in the main microorganisms involved in fermenting were evaluated. The indirect effect of moisture changes on maize silage fermentation was identified through the correlation analysis. Assessing the variability of maize silage fermentation at different moisture scales will provide further insights into microbial process under environmental changes.

## 2 Materials and methods

### 2.1 Plant material and experimental design

Four drought treatments were set up in this research: T1, drought treatment occurred at the seedling stage (V2–V10, including the third leaf from germination to maximum extension of the tenth leaf); T2, drought treatment at the elongation stage (V10–V12, the elongation stage of the nutritional growth phase); T3, drought treatment at the flowering stage (V12–R1, the 12-leaf spike to tassel stage); T4, drought treatment at maturity (R1–R3, from seed establishment to its one-half milking stage). The duration of drought stress at each period was 15 days. The control treatment was regular irrigation in the whole growth period of maize. Two moisture gradients were set up in the study (standard: GB/T 32136-2015). Severe drought: field water holding capacity about 40%; regular irrigation: field water holding capacity about 60%. Select “Quchen-9” for maize seeds. The experiment was conducted in a greenhouse at Yunnan Agricultural University (average temperature: 28.5°C). Maize seeds were sown in plastic pots (upper diameter: 41 cm, lower diameter: 30 cm, height: 25 cm). Each pot was filled with 18 kg of red soil. The soil organic matter content was about 48 g/kg and the nitrogen content was about 2 g/kg. There were 20 replicates for each treatment.

**Figure 1:**
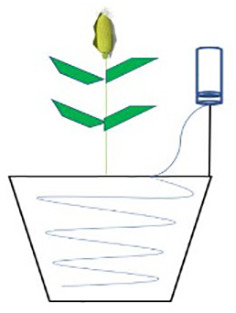
Experimental water control device.


Consistent with one of our previous studies (Zi et al., 2022), a self-made water control device was used for irrigation simulations (**Figure 1**). The irrigation method was referenced to a previous study (Buttler et al., 2019; Zi et al., 2022). Soil weight water content (SWC) was converted according to a first-order equation. The specific amount of irrigation was derived from SWC and real-time soil volumetric water content (determined every 1 day). Apparatus: SPectrum, TDR-100, USA. The amount of irrigation water was supplemented according to the conversion result of SWC. According to the formula, Mv for severe drought was about 10.58%. The drought treatments T1, T2, T3 and T4 were irrigated at about 133 mL per day. The Mv for regular irrigation (CK) was about 19.62%. The control treatment irrigated about 500 mL per day. The total irrigation volume was 5.5×10^4^ mL (T1), 5.6×10^4^ mL (T2), 5.6×10^4^ mL (T3), 5.2×10^4^ mL (T4) and 7.2×10^4^ mL (CK), respectively. The original formula is as follows.

(1) $$FC=0.2579\times Mv+0.2582,\;R^{2}=0.9998,$$

(2) $$Rsm=\frac{FC}{FC_{Max}}\times 100\%=\frac{\left(0.2579\times Mv+0.2582\right)}{21.2\%}\times 100\%$$

Note: FC, field capacity; Mv, volumetric water content; Rsm, elative soil moisture; FCmax, the maximum field capacity. R^2^ is close to one, indicating a high degree of linear correlation.

### 2.2 Determination of physiological and nutritional parameters

At harvest, the 10 pots of nearly developed maize were selected to determine the dry matter, root-to- shoot ratio and moisture content. The biomass of individual plants is measured at harvest and dried to determine the dry weight of the maize. The moisture content (%) is the ratio of water content (g) to biomass (g) of a single maize plant. The moisture content is the difference between the fresh and dry weight. The root-to-shoot ratio is the ratio of the fresh weight of the belowground and aboveground parts of maize. Maize leaves were sampled, dried, and ground to determine the soluble sugars, crude protein, and malonaldehyde content of the leaves. The top leaves of the maize were sampled and each treatment was replicated 3 times. The soluble sugar content was determined using the anthranilate method (Ramiro et al., 2016). The malonaldehyde content was measured using the thiobarbituric acid color reaction to assess membrane lipid peroxidation (Akhzari and Pessarakli, 2016). Nutritional parameters were measured once before and once after fermentation. The crude protein content was determined using an automated Kjeldahl nitrogen tester (Figueroa et al., 2002). The fat content was determined using an automatic fat tester (SZF-06C, China). The ash content was determined using the crucible scorch method (Wood et al., 1994). Neutral and acid detergent fibers were determined using a fiber tester (HN-F800, China). Calculation of RFV based on previous studies (Gheller et al., 2021). The equation for RFV is shown as follows.


(3) $$\;RFV=\frac{\left(DDM\times DMI\right)}{1.29}=\frac{\left(88.9-0.779ADF\right)\times \left(120÷NDF\right)}{1.29}$$

Note: RFV, relative feed value; NDF, neutral detergent fiber; ADF, acid detergent fiber.

### 2.3 Determination of silage quality

All maize was harvested and dried for 24 h. After drying, the maize tissue was cut evenly into small pieces (1–2 cm), samples were placed in polyethylene silage bags (350×450 mm, capacity: 2.5 kg), drained of air using a vacuum machine (RX-P290, Shanghai, China) and sealed with a sealer (RX8814, Shanghai, China); the samples were stored at room temperature (25°C). Fermentation was conducted for 60 days, sealed, and protected from light. All groups were repeated 3 times. Samples were taken once at each of the 3 different depth parts in each replicate and mixed. After the completion of fermentation, we sampled each group separately and measured the organic acids, pH, ammonia nitrogen, and aflatoxin content (Ding et al., 1997).

The pH was tested with a Sartorius PB-10 acidity meter (Sartorius Scientific Instruments, Beijing, China); the lactic acid content was measured with a high-performance liquid chromatograph (Thermo, U3000, USA); the acetic and propionic acid content was measured with a gas chromatograph (Shimadzu GC-2010, Japan). Operation procedure: Column: Agilent C18 AQ 4.6 mm*250 mm, 5 μm; column temperature: 35°C; injection volume: 10 μL; flow rate: 0.5 mL/min; wavelength: 210 nm; mobile phase: 10 mM K2HPO4 (pH=2.55). The concentration of ammonia nitrogen was determined using the indophenol blue method. Aflatoxin was determined using LC-MS (instrument, Agilent, 1290-6460); column: Agilent C18 (2.1 mm*100 mm, 3 μm); column temperature: 35°C, flow rate: 0.3 mL/min; acquisition mode: ESI+; injection volume: 5 μL.

### 2.4 Quantitative determination of microorganisms

In this research, the populations of 4 crucial microorganisms that influence feed quality during fermentation were measured. Beneficial bacteria: *Lactobacillus*; harmful bacteria: Molds and *E. coli*. Additionally, the total number of bacteria was determined. The microorganisms was determined using the plate count method (Ni et al., 2015). A sample of 10 g of each group was collected, added to 90 mL of saline, and shaken in a shaker. Each treatment was repeated 3 times. The mixed bacterial solution was gradually diluted and spread on MRS agar medium plates; 48 h of incubation at 37°C was used to count the *lactobacillus*. The total bacterial count and *E. coli* count were determined in a similar way. The bacterial solution was evenly spread on plates of potato dextrose agar medium, and molds were counted after incubation at a constant temperature of 30°C for 48–72 h. The units for total bacteria, lactic acid bacteria, and mold count: CFU/g. *E. coli* concentration in MPN/g.

### 2.5 Statistical analysis

Statistical analyses were performed using SPSS Statistics. 25 and Microsoft Excel 2010. Graphing was done with GraphPad Prism 8.0 and Bio-ladder Cloud. Statistical analyses of physiological indicators and fermentation quality were conducted using one-way ANOVA (Duncan’s method). Statistical analysis of nutritional parameters before and after fermentation was performed by two-way ANOVA (Friedman’s method). The correlation heat map is based on Pearson’s coefficient.

## 3 Results

### 3.1 Phenotypic and physiological characteristics under drought

**Figure 2:**
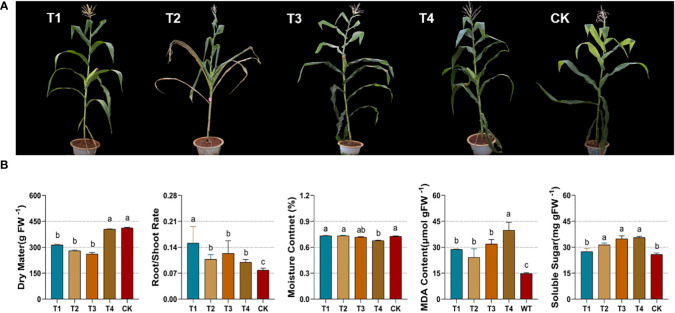
Phenotypes **(A)** and physiological parameters **(B)** of silage maize under different periods of drought treatments. **Figure 1B** represents from left to right, the changes in dry matter, root-shoot ratio, moisture content, malondialdehyde and soluble sugars. CK, control group; T1, seedling stage; T2, elongation stage; T3, flowering stage; T4, maturity stage. Different letters above the bars indicate that the values differ significantly (p<0.05).

Prolonged drought stress from the soil restricted the growth of maize silage throughout its development. In terms of plant morphology, the leaves of the maize group under drought were yellowed and curled, while the entire plant stalk and leaf volume were small (**Figure 2A**). The reduced area of light radiation and the closure of stomata on the leaf surface reduced the photosynthetic performance of green cells and the production of organic matter. As shown in **Figure 2B**, plant dry matter accumulation was significantly reduced (*p*<0.05) in T1, T2, and T3. The root-to-shoot ratio of the plants was a marker of response to their exposure to drought. The root-to-shoot ratio was significantly higher (*p*<0.05) in all drought groups (T1, T2, T3, and T4). The reduction in root water uptake during drought was accompanied by a significant decrease in the internal water content of the maize. The T4 group significantly reduced (*p* < 0.05) the moisture content of maize under drought, while the rest of the groups showed no significant changes. Malonaldehyde (MDA), which responds to the degree of damage to maize cell membranes, was also significantly increased (*p*<0.05) under alternative forms of drought. Drought stress leads to an overload of osmotic stress in maize leaf cells. To prevent cell death by water loss, an increase in free soluble sugar (SS) maintains plant cell homeostasis. Compared to the control, soluble sugar was significantly higher (*p*<0.05) in the maize group at the elongation, flowering, and maturity stages (**Figure 2B**). Drought at various periods resulted in a reduction in the dry matter of maize. Besides, there was a significant reduction of the moisture content under drought at the maturity stage. Drought stress increased the root-to-shoot ratio and in-plant malonaldehyde (MDA) and soluble sugar (SS) content.

### 3.2 Nutritional quality and relative feeding value of maize under PDS

**Figure 3:**
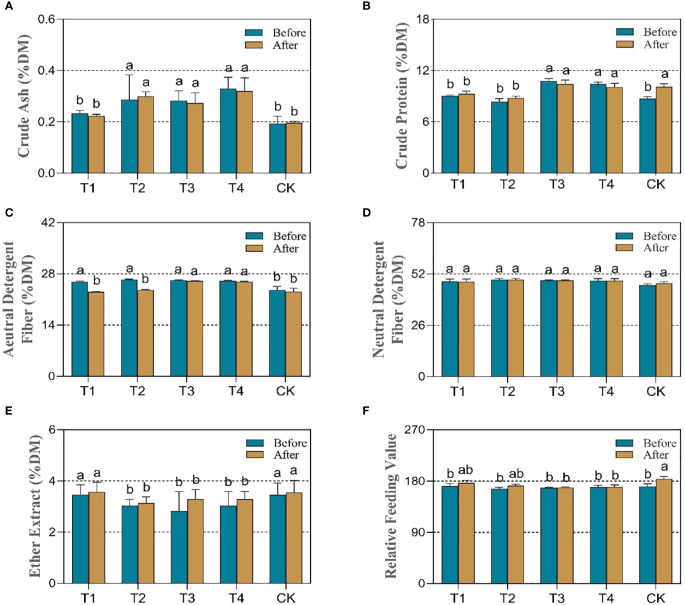
Effect of different periods of drought treatment on the dynamic nutritional quality of maize before (blue bar) and after treatment (yellow bar). The figure includes crude ash **(A)**, crude protein **(B)**, acid detergent fiber **(C)**, neutral detergent fiber **(D)**, ether extract **(E)**, and relative feeding value **(F)**. T1, seedling stage; T2, elongation stage; T3, flowering stage; T4, maturity stage. Different letters above the bars indicate that the values differ significantly (p<0.05).

Before silage fermentation, the critical nutritional parameters were evaluated to explore the response of maize quality to unique periods of drought stress. As shown in **Figure 3A**, drought at various stages significantly elevated the ash levels as inorganic salt ions that are detrimental to bodily health. In particular, drought stress at the elongation (T2), flowering (T3), and maturity (T4) stages resulted in 47.4%, 47.2%, and 73.7% significantly increases (*p*<0.05) in the percentage of ash. Simultaneously, the crude protein (CP) was significantly increased (*p*<0.05) by 24.1% and 19.8% in the drought groups T3 and T4, respectively, compared to the control (CK). However, no significant changes were observed in the T1 and T2 groups (**Figure 3B**).

Furthermore, the results (**Figure 3E**) showed that, except for the T1 group (seedling drought), the ether extract (EE) was significantly reduced (*p*<0.05) by 13.8%, 22.3%, and 13.8% for the remaining drought treatments, T2, T3, and T4, respectively. Under the four developmental periods of drought, acidic detergent fiber (ADF) demonstrated a significant increase (*p*<0.05) compared to CK (**Figures 3C, D**) and ranged between 9.61% and 11.85%. However, there was no significant difference in neutral detergent fiber (NDF) between groups. To further assess feed quality, the relative feeding value (RFV) was calculated to reflect the differences between groups (**Figure 3F**). The results showed drought decreased the relative feeding value (RFV) of the silage maize but did not reach a significant level. The value decreased by 2.5%, 1.6%, and 1.0% in the T2, T3, and T4 groups, respectively. It is clear that water stress exerts many negative effects on the nutritional quality of silage maize and that drought stress between the beginning of the elongation stage and maturity resulted in significant changes in the nutritional parameters. However, a certain degree of drought will enhance the crude protein content.

The results after fermentation showed no significant changes in the crude ash (ASH), ether extract (EE) and neutral detergent fiber (NDF) in the silage compared to before fermentation. The crude protein content in the CK group was significantly higher (*p* < 0.05) than that before fermentation by 16.1%, while there was no significant change in the drought group, and the crude protein content in the T1 and T2 drought groups was significantly lower (*p*<0.05) than that in control. In addition, the acid detergent fiber (ADF) content in T1, T2, and CK decreased significantly (*p*<0.05) after fermentation by 10.8%, 10.6%, and 5.1%, respectively, and its content in the T3 and T4 groups was still higher than that in control. The relative feeding value (RFV) was somewhat elevated in all groups due to the change in acid detergent fiber; however, the results in T3 and T4 groups was significantly lower (*p*<0.05) than that in CK.

### 3.3 Fermentation quality of silage

**Figure 4:**
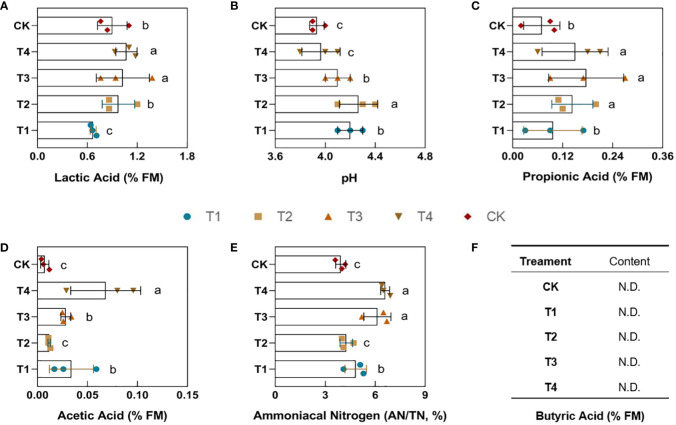
Effect of different periods of drought treatment on the fermentation quality of maize silage. The figure includes lactic acid **(A)**, pH **(B)**, propionic acid **(C)**, acetic acid **(D)**, ammoniacal nitrogen **(E)**, and butyric acid **(F)**. T1, seedling stage; T2, elongation stage; T3, flowering stage; T4, maturity stage. Different letters above the bars indicate that the values differ significantly (p<0.05).

After 60 days of silage fermentation, this study measured the percentage of lactic acid (LA), acetic acid (AA), propionic acid (PA), butyric acid (BA), pH, and ammoniacal nitrogen (AN) in the finished silages. Under drought stress, the lactic acid content in the feed was significantly (*p*<0.05) decreased by 0.234% in the T1 group compared to the control group. However, the lactic acid content increased to different degrees in the T2, T3, and T4 periods (**Figure 4A**). In particular, its content increased by 0.119% and 0.168% in the T3 and T4 groups, respectively, and reached a significant level (*p*<0.05). Drought caused the propionic acid content to increase to varying degrees as well. As be seen from **Figure 4C**, except for the non-significant difference in group T1, the propionic acid percentage was significantly (*p*<0.05) higher in groups T2, T3, and T4 by 0.07%, 0.11%, and 0.08%, respectively, compared to CK. The content of acetic acid did not change significantly in the T2 treatment compared to the control CK. However, the percentage of the acetic acid was significantly higher (*p*<0.05) in the T1, T3, and T4 groups by 0.03%, 0.06%, and 0.06%, respectively (**Figure 4D**). Ultimately, our results showed that no butyric acid was detected in any of the feeds (**Figure 4F**), which may indicate that the feed was healthy.

The pH of the drought groups increased to varying degrees compared to the control as organic acids changed (**Figure 4B**). The T1, T2, and T3 groups showed significant (*p*<0.05) increases of 0.27%, 0.34%, and 0.17%, respectively, while treatment T4 demonstrated a minor increase of 0.04% with no significant change. The ammoniacal nitrogen (AN) typically represents the degree of protein breakdown in the feed. Additionally, its content was measured and assessed the loss of nutrients (**Figure 4E**). The results showed that the percentage of ammoniacal nitrogen was significantly higher (*p*<0.05) in groups T1, T3, and T4 by 1.7%, 1.6%, and 2.8%, respectively. The drought treatment T2 revealed no significant change in ammoniacal nitrogen. Different periods of drought did not lead to changes in butyric acid in fermentation. However, drought led to a significant decrease in lactic acid and an increase in acetic acid, pH, and ammoniacal nitrogen during the T1 period. Secondly, the lactic acid in maize feeds did not change significantly during the T2 period, and propionic acid and pH increased significantly. In addition, the lactic acid in the feed during the T3 period increased significantly, while the propionic acid, acetic acid, pH, and ammoniacal nitrogen indicated a significant upward trend. Ultimately, the lactic acid content of the silage in the T4 period increased significantly, the propionic acid, acetic acid, and ammoniacal nitrogen contents are also significantly increased.

### 3.4 Analysis of microorganisms and mycotoxins in maize fermentation

**Figure 5:**
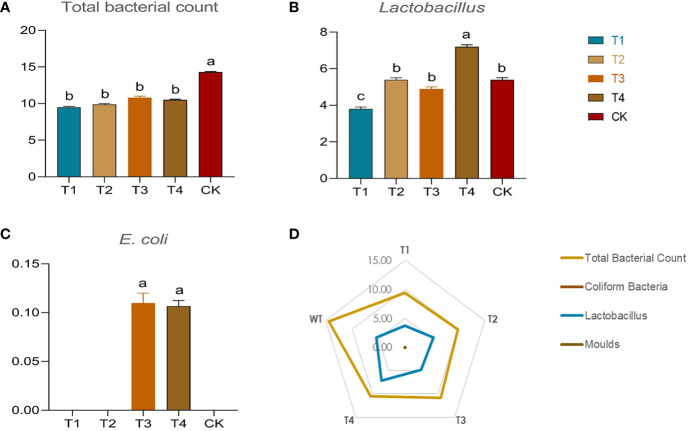
Bacterial counts and proportions in different drought treatments and controls. The graph includes **(A)** total bacterial counts, N*10^7^ CFU/g; **(B)** *lactobacillus* counts, N*10^7^ CFU/g; **(C)** *E coli* counts, N*10^7^ MPN/g; **(D)** summary radar plots. The N in front indicates the number in the figure. The numbers in the graph indicate the specific bacterial content of each group.

In this study, the population number of main microorganisms involved in the fermented silages were quantified (**Figure 5D**). Different periods of drought resulted in significant changes in the number of *lactobacillus* communities in the fermentations (**Figure 5B**). Only in the T4 period did drought directly and significantly increased the number of *lactobacillus* populations (7.20*10^7^ CFU/g), by a significant (*p*<0.05) value of 33% over the control group (5.40*10^7^ CFU/g). In contrast, the percentage of *lactobacillus* significantly decreased (*p*<0.05) by 31.4% and 10.2% in the T1 and T3 groups, respectively. Focusing on total bacterial counts, the total bacterial community counts were significantly higher in fermentations based on conventionally irrigated (CK) maize than in the other drought treatments (**Figure 5A**). Drought may have reduced bacterial diversity in fermentation, which warrants more isolated exploration. The E*. coli* counts increased by 1% in both the T3 and T4 groups (**Figure 5C**) compared to the control but did not change significantly in the T1 and T2 groups. In addition, the amount of mold in the silage was negligible (<1 CFU/g). Moreover, the aflatoxin-B1 and aflatoxin-B2 were not detected in the silage maize, which may indicate that drought did not lead to significant aflatoxin colonization in the silage. The silage fermentation under drought may be relatively healthy.

### 3.5 Correlation analysis of nutritional and silage quality parameters

**Figure 6:**
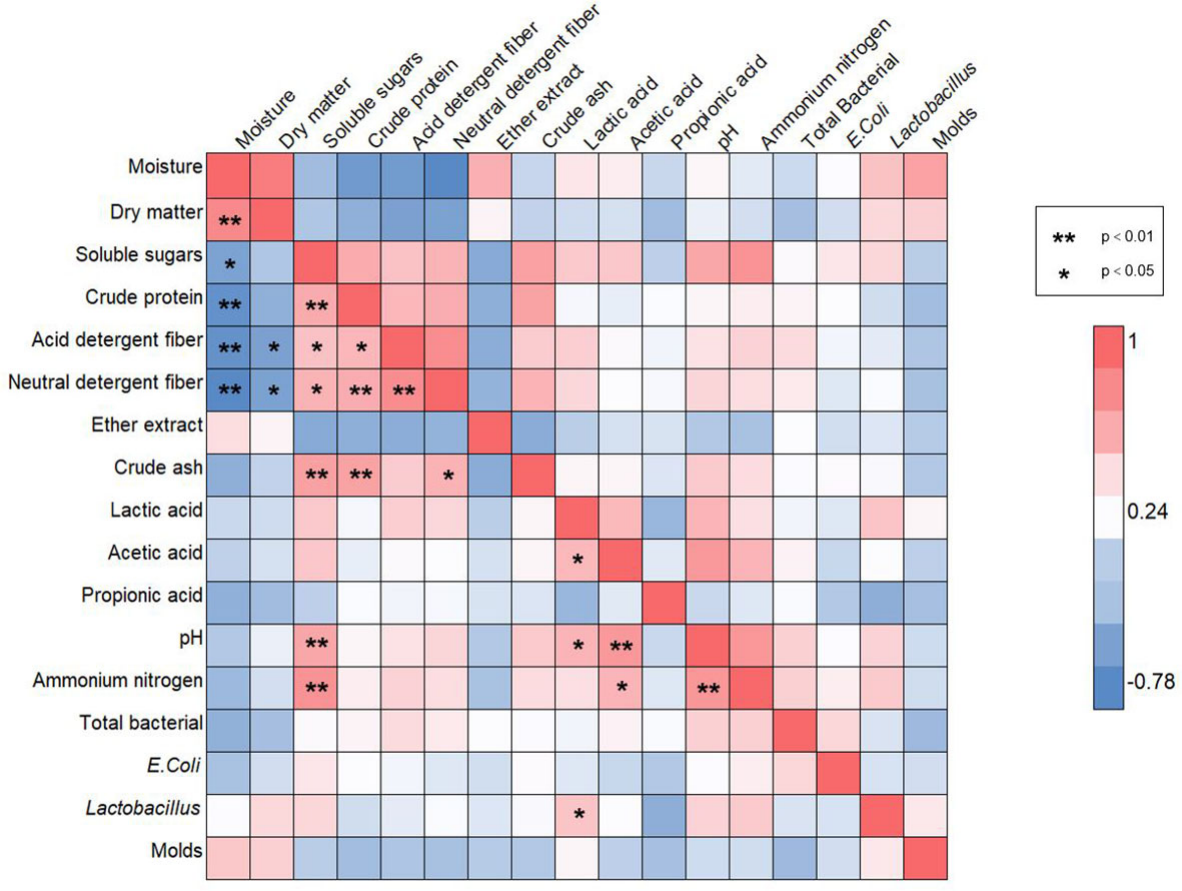
Pearson correlation heat map for all chemical components or microorganisms. The “*” in the graph indicates a significant correlation between indicators at the *p* < 0.05 level, while “**” indicates a significant correlation between indicators at the *p* < 0.01 level. In the graph, blue squares represent negative correlations, while red squares represent positive correlations.

Pearson correlation analysis was performed on 17 indicators (**Figure 6**). These indicators include physiological parameters, nutritional parameters, silage quality and microbial counts. Based on the results, the beneficial or negative effects of the quality indicators under drought stress were assessed. The moisture content of the silage material indicated a significant positive correlation only for the dry matter. However, moisture content showed significant negative correlations for the soluble sugars and the nutritional qualities (crude protein, acid detergent fiber and neutral detergent fiber). In contrast, soluble sugars demonstrated significant positive correlations for the nutritional parameters (crude protein, acid detergent fiber, neutral detergent fiber, crude ash) and significant negative correlations for the fermentation qualities pH and the ammonium nitrogen content. In addition, the percentage of lactic acid after fermentation was significantly positively correlated with pH and the ammonium nitrogen content. Of the microbial community counts, only lactobacillus is significantly positively correlated with the lactic acid content. As primary decomposers, other microorganisms such as *lactobacillus* have difficulty in using starch efficiently. As a result, maize exposed to drought affected the rapid multiplication of microbial populations during anaerobic fermentation, which eventually led to changes in silage quality.

Based on the results of the correlation analysis, a scientific hypothesis is proposed: drought regulates the process of maize silage fermentation. Drought-stricken maize compressed the space for molds in fermentation and that microbial diversity is reduced. Interestingly, despite the concomitant decrease in dry matter accumulation and increased protein decomposition, the survival of *lactobacillus* was significantly higher under prolonged drought at maturity and there was some increase in lactic acid production. In brief, water stress during late maize growth may indirectly regulate the fermentation in the micro-ecosystem and influence its function.

## 4 Discussion

With changes in the form of soil drought, drought stress at various times has an impact on agricultural production and livestock (Chen et al., 2022). The effective combination of microbiology and crop science has better interpreted the changes in physiological metabolism and ecological functions of drought-affected plants (Simonin et al., 2020). To date, only a relatively limited number of studies have focused on changes in the fermentation of silage from drought-stricken crops. This study measured the quality parameters and microbial community population of silages. By correlating the metabolic changes of silage maize under drought with its fermentation products and bacterial changes, this research illustrated the effectiveness of drought environment regulation of maize silage quality.

Plant phenotypes can reflect their intuitive changes under abiotic stress. This study discovered significant changes in maize phenotypes under drought at the seedling and elongation stages. The yellow leaf coloration can be attributed to the significant accumulation of flavonoids in leaf cells (Geng et al., 2020). Under drought stress, the reduced photosynthetic performance of maize accompanied by reduced leaf area leads to insufficient accumulation of dry matter, as has been likewise found in rice and wheat (Foulkes et al., 2007; Cox, 2022). Drought-stricken maize also accumulates malonaldehyde in large amounts, which allows the assessment of the extent of cell membrane damage (Fukao et al., 2011). The results of our study are in agreement with this. Similarly, the increases of soluble sugars accumulation in active plant cells to regulate osmotic pressure homeostasis under conditions of water loss (Barrios-Masias et al., 2015). In addition, the maize root system is more developed under drought, with a consequent increase in the root-to-shoot ratio caused by the decrease in photosynthetic assimilates, in relative terms. The decrease of moisture content is additionally an indication that plants were suffering from drought (Burnett et al., 2021). A previous study of ours noted (Zi et al., 2022) that drought stress during the flowering stage of maize significantly reduced the biomass. This was confirmed by the reduction in dry matter in the present study. Despite the fact that drought stress at post developmental stages (flowering and harvest stages) significantly increased the crude protein content, the yields and dry matters significantly reduced under water stress. Under long-term adversity stress, drought affects the structural composition of polysaccharide molecules and therefore may cause an indirect effect on other quality parameters. For macromolecular organic matter, the maize dry matter was significantly decreased under moisture stress, whereas the crude protein content was significantly elevated (Zi et al., 2022). Drought stress accelerates the process of lignification and senescence in maize. It was noted that the fiber content was significantly increased in drought-stressed variants of cotton (Wenqing et al., 2019). It has been documented that water stress increases the drought tolerance of the cell wall (Geng et al., 2018). Drought causes an enrichment of the phenylpropane metabolic pathway, associated with the synthesis of lignin (Vincent et al., 2005). The increase in acid detergent fiber may be due to the accelerated lignification of maize cells by drought. Therefore, a large number of polysaccharides is involved in the composition of mechanical structures in maize tissues, a result that causes a decrease in feed palatability for animals (He et al., 2020a). Our results indicated that water stress had a range of negative effects on the nutritional quality of silage maize; drought stress during the period from the beginning of elongation to maturity resulted in significant changes in nutritional parameters. A certain degree of drought stress increases the crude and beneficial protein content for silage, as well as the acid detergent fiber content. It is noteworthy that few studies have been conducted on drought-induced changes in fermentation quality. One research noted that fermentation of drought-stressed maize variants resulted in a significant increase in pH and reduced digestibility of organic matter as well as the ammonia-nitrogen concentration (Meibaum and Breves, 2012). The results of this study showed that drought caused a significant decrease in lactic acid during the seedling period but was accompanied by an increase in acetic acid, ammoniacal nitrogen and the pH value. Meanwhile, the result shows that alternative forms of drought did not lead to changes in butyric acid in fermentation. In contrast, for silage fermentation, the maize subjected to drought during the filling period significantly elevated the number of the *lactobacillus* and lactic acid content. Drought-affected maize showed a significant increase in lactic acid in fermentation accompanied by a significant upward trend in acetic acid, propionic acid and the ammoniacal nitrogen content under drought at the flowering and maturity stages.

The reduction of moisture also implies that less water is present for the microbial community during fermentation, which may the reason for the impact on material cycling in silage fermentation and the reduced ability of mold colonization (Da Silva et al., 2019). Similar conclusions have been drawn in soil microbial studies (Erlandson et al., 2016). A previous study showed that the mild water stress can change the diversity and richness of soil microorganisms in greenhouse grape soils (Zhang et al., 2019). Repeated droughts increase the differences in microbial communities (Canarini et al., 2021). Consistent with the fermentation process, in ecology, plants and microorganisms interact (Thomsen et al., 2022). Simultaneously, a study points out that plants define the microbial society in the inter-rhizosphere soil, especially with the strongest impact on fungal colonization and bacterial diversity (Kuzyakov and Blagodatskaya, 2015). This suggests that the maize inner substance produce a typical shaping of the fermentation microbial community (He et al., 2020b). Drought disrupts the homeostasis of the fermentation microenvironment to which the bacterial community responds. Correspondingly, a significant decrease was observed in total bacterial and mold counts in the present study. Moreover, the *lactobacillus* are the main beneficial decomposers of silage fermentation, and their product lactic acid is one of the key indicators for determining the feed quality (Chen et al., 2021). It’s worth note that high levels of lactic acid led to an increase in pH, and an acidic environment would be detrimental to the survival of spoilage bacteria (Gallo et al., 2022). For example, a previous study indicates the pH value can affect molds growth (Kia et al., 2018). The results of this study show that the lactic acid content was elevated in fermentations under drought during flowering and maturity of maize.

The moisture content of the raw material prior to fermentation and soluble sugars are key microbial regulators (Zeng et al., 2020). Although the correlation between the two in terms of fermentation quality was not significant, they did indicate a positive correlation with the fermentation quality parameters. Drought at flowering and maturity stages resulted in a significant decrease in the water content and a significant increase in soluble sugars (He et al., 2020c). This leads to an alteration in the nutrients required for microbial reproduction in fermentation (Mu et al., 2020). In addition, there was a significant increase in total *lactobacillu*s count in the finished feed after silage fermentation in both groups. The contents of lactic acid, acetic acid, and ammonium nitrogen were significantly increased in the group of maturation drought. Drought caused drastic changes in the environmental factors of the fermentation system, which led to nutrient and water heterogeneity in the microbial community (Fernandes et al., 2021). Indeed, drought also alters the flow of energy and simple material exchange in the microbial ecosystem (Ma et al., 2022). Differences in the moisture content and soluble sugars of individual maize led to differences in the energy allocation of the bacterial community and thus to microbial interspecific competition. These changes reshape the micro-ecosystem of silage anaerobic fermentation (Yuan et al., 2022). Simultaneously, drought significantly affected feed quality. Drought stress at maturity stage brought positive and negative effects on silage maize. A brief and middle drought (Rsm>50%, rain avoidance) before harvest might be able to increase the lactic acid and crude protein content within a certain range. Meanwhile, it may maintain the sustainability and nutritional value of silage fermentation with a minor loss of fresh and dry weight. Furthermore, the early drought stress in development (seedling and elongation stages) should be avoided in maize cultivation production practices by using drip irrigation or other methods to maintain continuous adequate moisture. For mild droughts just before harvest, perhaps the post-mowing drying process of silage maize could be omitted to prevent excess plant water content. These findings will facilitate more isolated improvements in feedstock management practices and provide a theoretical basis for related research.

## 5 Conclusion

This study characterized changes in the physiological and nutritional parameters as well as the fermentation quality and microorganisms in maize exposed to drought. Drought significantly reduced maize dry matter and moisture but increased soluble sugars, the malonaldehyde content and root-to-shoot ratio. Water stress significantly elevated crude protein and acidic detergent fiber content while decreasing ether extract content. Furthermore, drought in maize at maturity significantly reduced the number of total bacteria and molds in silage fermentation but increased the proportion of the *lactobacillus* and the lactic acid content of silage maize. This change was beneficial for the silage production. However, drought also led to an increase in ammonia nitrogen content and pH value in the silage. Overall, maize silage subjected to environmental drought altered the microbial ecosystem during fermentation, reorganizing the bacterial community and metabolite diversity. Drought delivered both beneficial and negative effects on the silage fermentation of maize.

## Data availability statement

The raw data supporting the conclusions of this article will be made available by the authors, without undue reservation.

## Author contributions

Funding acquisition, BW; Investigation, XZ, WW, SZ, FZ, DR, PS, and SF; Methodology, XZ, WW, SZ, FZ, DR, PS, and SF; Project ad-ministration, BW; Writing—original draft, XZ and WW; Writing review and editing, BW. All authors have read and approved the final manuscript.
